# Country-level land carbon sink and its causing components by the middle of the twenty-first century

**DOI:** 10.1186/s13717-021-00328-y

**Published:** 2021-09-14

**Authors:** Lifen Jiang, Junyi Liang, Xingjie Lu, Enqing Hou, Forrest M. Hoffman, Yiqi Luo

**Affiliations:** 1 Center for Ecosystem Science and Society, Northern Arizona University, Flagstaff, AZ 86011 USA; 2 College of Grassland Science and Technology, China Agricultural University, Beijing, 100083 China; 3 School of Atmospheric Sciences, Sun Yat-sen University, Guangzhou, 510275 Guangdong China; 4 Computational Sciences & Engineering Division and Climate Change Science Institute, Oak Ridge National Laboratory, Oak Ridge, TN 37831 USA; 5 Department of Biological Sciences, Northern Arizona University, Flagstaff, AZ 86011 USA

**Keywords:** Carbon sink, Carbon storage, Earth system models, Net primary productivity, Residence time, Terrestrial ecosystems

## Abstract

**Background:**

Countries have long been making efforts by reducing greenhouse-gas emissions to mitigate climate change. In the agreements of the United Nations Framework Convention on Climate Change, involved countries have committed to reduction targets. However, carbon (C) sink and its involving processes by natural ecosystems remain difficult to quantify.

**Methods:**

Using a transient traceability framework, we estimated country-level land C sink and its causing components by 2050 simulated by 12 Earth System Models involved in the Coupled Model Intercomparison Project Phase 5 (CMIP5) under RCP8.5.

**Results:**

The top 20 countries with highest C sink have the potential to sequester 62 Pg C in total, among which, Russia, Canada, USA, China, and Brazil sequester the most. This C sink consists of four components: production-driven change, turnover-driven change, change in instantaneous C storage potential, and interaction between production-driven change and turnover-driven change. The four components account for 49.5%, 28.1%, 14.5%, and 7.9% of the land C sink, respectively.

**Conclusion:**

The model-based estimates highlight that land C sink potentially offsets a substantial proportion of greenhouse-gas emissions, especially for countries where net primary production (NPP) likely increases substantially and inherent residence time elongates.

## Introduction

Climate change is a big threat to the whole world. The global mean surface temperature has increased by 1.0 °C since pre-industrial levels, which is mainly caused by human activities; and the anthropogenic global warming is still ongoing at a speed of 0.2 °C per decade (IPCC 2018). Great efforts have been made by scientists and governments both nationally and internationally to adapt to and mitigate climate change. The United Nations Framework Convention on Climate Change (UNFCCC) adopted in Rio de Janeiro, Brazil in 1992, which envisaged a significant reduction in emissions of greenhouse gases (GHGs) into the atmosphere, primarily carbon dioxide (СО_2_), was a historical start for the collective commitments by many countries to mitigate climate change (Akaev 2017). Following that, a big milestone was accomplished in 1997 in Kyoto, Japan, i.e., the Kyoto Protocol. In the Kyoto Protocol (https://unfccc.int/resource/docs/convkp/kpeng.pdf), it was specified the reduction of СО_2_ emissions for many countries of the world. At the 21st Conference of the Parties (СОР 21) to the UNFCCC, held in December of 2015 in Paris, a new climate agreement, the Paris Agreement, was adopted by many countries to replace the Kyoto Protocol after 2020 for continuous efforts to mitigate climate change. The target of the Paris Agreement is “holding the increase in the global average temperature to well below 2 °C above pre-industrial levels and pursuing efforts to limit the temperature increase to 1.5 °C above pre-industrial levels, recognizing that this would significantly reduce the risks and impacts of climate change” (https://unfccc.int/process/conferences/pastconferences/paris-climate-change-conference-november-2015/paris-agreement).

Recognizing the dramatic differences in the impacts and risks for selected natural, managed, and human systems between 1.5 and 2 °C warming, the special report of IPCC (2018), Global Warming of 1.5 °C, has provided different 1.5 °C-consistent emission pathways to limit warming either below 1.5 °C, or returning to 1.5 °C by around 2100 following an overshoot. These pathways, as well as those emission reductions in the Kyoto Protocol and the Paris Agreement, however, are primarily built upon a rapid phase out of CO_2_ emissions and deep emission reductions in other GHGs and climate forcers through broad transformations in the energy, industry, transport, buildings, Agriculture, Forestry, and Other Land-Use (AFOLU) sectors. Carbon (C) sequestered by natural terrestrial and ocean ecosystems is not well quantified to act as a critical C sink to offset a proportion of the anthropogenic emissions.

Indeed, natural terrestrial and ocean ecosystems can sequester substantial CO_2_ from the atmosphere each year. For example, the terrestrial ecosystems are estimated to take up 3.0 ± 0.8 Gt C year^−1^, approximately one-third of the CO_2_ emissions from fossil fuels and industry (Le Quéré et al. 2018; Friedlingstein et al. 2019). Forests can play a key role in meeting climate targets in the Paris Agreement by providing a quarter of emission reductions committed by countries and turning the globe from a net anthropogenic C source during 1990–2010 to a net C sink by 2030 (Grassi et al. 2017). Therefore, the C sink from natural terrestrial and ocean ecosystems needs to be taken into account as an important component when different countries make their policies to reach net zero or negative emissions in order to limit global warming to 1.5 °C.

Scientists have long been exploring C sink of the natural ecosystems both on land and in Ocean. Carbon sink of various terrestrial ecosystems has been widely studied at local or regional scale in the context of mitigating climate change (Smith et al. 2005; Tan and Lal 2005; Niu and Duiker 2006; Grelle et al. 2007; Kaul et al. 2010; Kongsager et al. 2013; Zhou et al. 2015). Carbon sequestration by terrestrial ecosystems in response to climate change in the future has also been predicted with statistical models, process-based ecosystem models, or land surface models, commonly showing a considerable global land C sink potential (Espinosa et al. 2005; Smith et al. 2005; Friedlingstein et al. 2006, 2014; Jones et al. 2013; Hararuk et al. 2014; Tan et al. 2015; Chazdon et al. 2016). However, to date, there are no such studies on land C sink and its causing components at the country level.

Carbon sink is usually investigated as changes in C storage or as flux that C enters into an ecosystem, often known as net ecosystem production (NEP) or net ecosystem exchange (NEE). Both changes in C storage or C influx to an ecosystem are C dynamics in response to external climate forcing, land use change or succession, so-called transient C storage or C flux. In terms of removing CO_2_ from the atmosphere in the long run, it is important that we know not only how much C that an ecosystem or the globe can potentially sequester, but also what component(s) dominates this C sink. Yet, the causing components, or in other words, the involving processes, of land C sink have not been well explored. Using a linearization approach, Koven et al. (2015) separated C storage changes in response to doubled atmospheric CO_2_ of the live and dead carbon pools simulated by five Earth system models (ESMs) involved in the Coupled Model Intercomparison Project Phase 5 (CMIP5) into input-related changes, i.e., productivity-driven changes and output-related changes, i.e., turnover-driven changes. More theoretically or mechanically, transient C storage of any ecosystem at a time has been quantified as the difference between instantaneous C storage capacity and instantaneous C storage potential by Luo et al. (2017). In detail, C storage capacity is the maximum amount of C that an ecosystem can store at any given time and it is the product of net primary production (NPP) and C residence time. Instantaneous carbon storage potential represents the internal capability of an ecosystem to equilibrate the current C storage with the C storage capacity. It indicates a potential of an ecosystem to store additional C when it has a positive value or a potential to lose C when the value is negative at a given time. This new framework that decomposes transient C storage into instantaneous C storage capacity and instantaneous C storage potential has an advantage over the traditional ways in investigating dynamics of C storage because it takes into account the inherent characteristics of an ecosystem, such as NPP and C residence time. NPP and C residence time are the most important components that dominate uncertainty in terrestrial vegetation and soil C responses to climate and atmospheric CO_2_ (Todd-Brown et al. 2013; Friend et al. 2013; Jiang et al. 2015).

The theory of transient C storage developed by Luo et al. (2017) has been applied to explore the mechanisms behind the dynamic of the transient C storage in response to climate change among different ecosystems (Jiang et al. 2017) or among different ESMs (Zhou et al. 2018). In this study, we analyzed model output of 12 ESMs involved in CMIP5 based on this theoretical decomposition of transient C storage. The objective is to quantify the land C sink and its causing components of different countries by the middle of the twenty-first century under the most severe climate change scenario with a new framework and therefore, to help policy makers to better mitigate climate change.

## Materials and methods

### The transient traceability framework for carbon storage dynamics

In this study, land C sink of different countries by 2050 was explored with a transient traceability framework for C storage dynamics with CMIP5 model output. Carbon sink refers to the difference in C storage between two time steps, i.e., year 2050 (average of 2046–20250) and year 2005 (average of 2001–2005), respectively.

The transient traceability framework for C storage dynamics was first theoretically analyzed by Luo et al. (2017) based on the fact that most land C cycling models shared some common properties. It was then applied to two forest ecosystems to trace the different responses of C storage to climate change and to reveal the mechanisms underlying the different responses (Jiang et al. 2017). In this traceability framework, transient C storage at a certain time is jointly determined by instantaneous C storage capacity and instantaneous C storage potential as expressed by the following equation:

1 $$ X(t)={X}_c(t)-{X}_p(t) $$

where *X*(*t*) is transient C storage; *X*_*c*_ is C storage capacity, which is defined as the maximum instantaneous C storage without any environmental or other restrictions at a given time; and *X*_*p*_ is instantaneous C storage potential, which represents the unrealized C storage due to environmental or other limitations at a given time.

Furthermore, C storage capacity, *X*_*c*_, is co-determined by C input (gross fv, *τ*(*t*), as shown in the following equation:

2 $$ {X}_c(t)= NPP(t)\times \tau (t) $$

And instantaneous C storage potential, *X*_*p*_, is a product of net C pool change, *X'*(*t*), and chasing time. Chasing time measures the time needed for net C pool change to be redistributed in the network with all C pools, which is not further explored in this analysis.

### CMIP5 model output

We used model output of historical simulations and representative concentration pathway (RCP) 8.5 of 12 ESMs in CMIP5, including bcc-csm1-1-m, BNU-ESM, CanESM2, CESM1-BGC, GFDL-ESM2G, HadGEM2-ES, inmcm4, IPSL-CM5A-LR, MIROC-ESM, MPI-ESM-LR, MRI-ESM1, and NorESM1-ME. Details of these ESMs were given in Table 1. RCP8.5 represents the most severe climate change scenario among all scenarios. We downloaded model output of monthly C mass in all land C pools, including C in vegetation (variable “cVeg”), course wood debris (variable “cCwd”), litter (variable “cLitter”), and soil (variable “cSoil”). Carbon pools were then added together to get ecosystem C. Monthly NPP (variable “npp”) was also downloaded for decomposition of land C sink. For the inmcm4 model, we calculated its npp as the difference between GPP (variable “gpp”) and autotrophic respiration (variable “ra”) because this model did not have direct output of npp. Monthly C pools and NPP were converted to yearly data. In addition, land area fraction (variable “sftlf”) of each model was used for computing carbon storage and NPP of different countries.

**Table 1:** Summary of CMIP5 ESMs and their land carbon cycle components

Model name of ESMs	Version	Modeling group	Land carbon cycle components	Number of plant functional types	Number of live carbon pools	Dynamic vegetation	Nitrogen cycle	Original resolution of latitude and longitude (° lat × ° lon)	References
BCC-CSM1.1	20120918	Beijing Climate Center, China Meteorological Administration	BCC-AVIM1.0	15	3	No	No	2.81 × 2.81	Ji et al. 2008; Wu et al. 2013
BNU-ESM	20120504	College of Global Change and Earth System Science, Beijing Normal University	CoLM+BNUDGVM (based on LPJ)	10	4	Yes	No	2.81 × 2.81	Dai et al. 2003, 2004; Ji et al. 2014
CanESM2	20120410	Canadian Centre for Climate Modeling and Analysis	CLASS2.7+CTEM1	9	3	No	No	2.81 × 2.81	Arora and Boer. 2010
CESM1-BGC	20121029	Community Earth System Model Contributors	CLM4	15	4	No	Yes	0.94 × 1.25	Thornton and Zimmermann 2007; Thornton et al. 2007, 2009; Lawrence et al. 2011
GFDL-ESM2G	20121206	Geophysical Fluid Dynamics Laboratory	LM3	5	5	Yes	No	1.99 × 2.48	Shevliakova et al. 2009; Dunne et al. 2013
HadGEM2-ES	20111007	Met Office Hadley Centre	TRIFFID	5	3	Yes	No	1.24 × 1.88	Cox 2001; Collins et al. 2011; Jones et al. 2011; The HadGEM2 Development Team 2011
INM-CM4	20110323	Institute for Numerical Mathematics	LSM 1.0	12	3	No	No	1.50 × 2.00	Bonan 1996; Volodin 2007
IPSL-CM5A-MR	20120430	Institut Pierre Simon Laplace	ORCHIDEE	12	8	No	No	1.26 × 2.50	Dufresne et al. 2013; Krinner et al. 2005
MIROC-ESM	20120710	Japan Agency for Marine- Earth Science and Technology, Atmosphere and Ocean Research Institute (The University of Tokyo), and National Institute for Environmental Studies	MATSIRO+SEIB–DGVM	13	4	Yes	No	2.81 × 2.81	Sato et al. 2007; Watanabe et al. 2011
MPI-ESM-MR	20120503	Max Planck Institute for Meteorology	JSBACH	12	3	Yes	No	1.88 × 1.88	Raddatz et al. 2007; Brovkin et al. 2009; Reick et al. 2013
MRI-ESM1	20130307	Meteorological Research Institute	Models of the biochemical photosynthesis processes at leaf level and LPJ-DGVM at ecosystem level	10	3	Yes	No	1.00 × 0.50	Adachi et al. 2013
NorESM1-ME	20120225	Norwegian Climate Centre	CLM4	15	4	No	Yes	1.88 × 2.50	Tjiputra et al. 2013

For each model, we used the ensemble member r1i1p1 as it is the only ensemble available for all CMIP5 models. In addition, a previous study has demonstrated that simulations among the multiple ensembles from a single model were similar in general (Jiang et al. 2015). The letters “r,” “i,” and “p” in the ensemble name indicate the initial condition, initialization method and perturbed physics version, respectively and “1” after each letter is the realization number for the respective parameter (Taylor et al. 2010, 2012).

We analyzed land C sink of 12 ESMs at both grid level and country level and decomposed the land C sink into four components at country level as described in the next section. Land C sink was calculated as the C storage difference between year 2050 (average of 2046–2050) of RCP8.5 and year 2005 (average of 2001–2005) of historical runs. The country border map we used was from WorldClim (http://www.worldclim.org). Because the original resolutions of CMIP5 model outputs were different from one another (Table 1) and were also different with the country border map, we regridded C density and NPP of model outputs to match the resolution of the country border map using conservation of mass.

### Application of the transient traceability framework to CMIP5 model outputs

Land C sink, i.e., the difference in C storage between two time steps, *∆X*, can be achieved by the following equation according to Eq. 1:

3 $$ \Delta X=\Delta {X}_c-\Delta {X}_p $$

Change of C storage capacity (∆*X*_*c*_) is equal to:

4 $$ \Delta {X}_c={\mathrm{NPP}}_1\times {\tau}_1-{\mathrm{NPP}}_0\times {\tau}_0 $$

where NPP_1_ and *τ*_1_ are NPP and *τ* at time step 2, respectively, which is year 2050 in this study; and NPP_0_ and *τ*_0_ are NPP and *τ* at time step 1 (i.e., year 2005), respectively.

If we add and then minus a term, NPP_1_ × *τ*_0_, which does not change the equation, equation 4 becomes:

5 $$ \Delta {X}_c={\mathrm{NPP}}_1\times {\tau}_1-{\mathrm{NPP}}_1\times {\tau}_0+{\mathrm{NPP}}_1\times {\tau}_0-{\mathrm{NPP}}_0\times {\tau}_0 $$

Because the term “*τ*_1_ - *τ*_0_” is actually the change of *τ*(∆*τ*), and similarly, the term “NPP_1_ - NPP_0_” means the change of NPP (∆NPP), equation 5 can be changed to:

6 $$ \Delta {X}_c=\Delta \tau \times {\mathrm{NPP}}_1+\Delta \mathrm{NPP}\times {\tau}_0 $$

As explained above, NPP_1_ is sum of NPP_0_ and the change of NPP (∆NPP), so equation 6 can be written as:

7 $$ \Delta {X}_c=\Delta \tau \times \left({\mathrm{NPP}}_0+\Delta \mathrm{NPP}\right)+\Delta \mathrm{NPP}\times {\tau}_0 $$

And Eq. 7 can be reorganized into the following format:

8 $$ \Delta {X}_c=\Delta \mathrm{NPP}\times {\tau}_0+\Delta \tau \times {\mathrm{NPP}}_0+\Delta \mathrm{NPP}\times \Delta \tau $$

Combining Eqs. 3 and 8, we get the equation for deriving C sink (∆*X*):

9 $$ \Delta X=\Delta \mathrm{NPP}\times {\tau}_0+\Delta \tau \times {\mathrm{NPP}}_0+\Delta \mathrm{NPP}\times \Delta \tau -\Delta {X}_p $$

With Eq. 9, we can trace land C sink into four components, i.e., production-driven change (∆NPP × *τ*_0_), turnover-driven change (∆*τ* × NPP_0_), interaction between production-driven change and turnover-driven change (∆NPP × ∆*τ*), and change in instantaneous C storage potential (∆*X*_*p*_). Initial NPP and initial *τ* here represent values of NPP and *τ* at time step 1, i.e., year 2005 in specific. In this study, we calculated *τ* and *X*_*p*_ using the method adopted by Zhou et al. (2018):

10 $$ \tau =\frac{X}{NPP-{X}^{\prime }} $$

11 $$ {X}_p(t)={X}_c(t)-X(t) $$

**Fig. 1:**
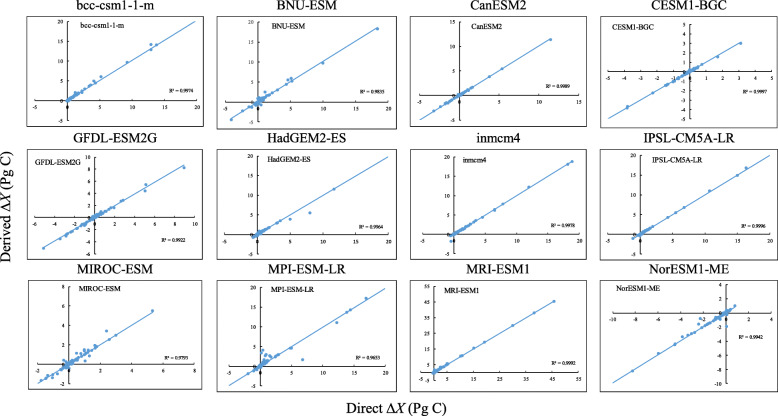
Validation of land carbon sink (∆*X*) calculations with the transient traceability framework. Derived ∆*X* is calculated by Eq. 9 and direct ∆*X* is the difference in carbon storage between the average of 2046–2050 and the average of 2001–2005 calculated directly from CMIP5 model outputs

We calculated yearly *X*′, *τ*, *X*_*c*_, and *X*_*p*_ at both grid level and country level. For both levels, we averaged results from 2001 to 2005 to represent year 2005 and the results from 2046 to 2050 to represent year 2050. At grid level, we excluded *τ* greater than 500 years before outputting the results because most previous studies have shown that global averaged ecosystem residence time is much less than 100 years (Carvalhais et al. 2014; Lu et al. 2018; Wu et al. 2020). At country scale, we first regridded ecosystem C and NPP of each model to the resolution of the country border map after multiplying by land area fraction. Then we could calculate ecosystem C storage and NPP of countries. After that, *X*′, *τ*, *X*_*c*_, and *X*_*p*_ of countries for each model were calculated. Within each model, if a country had 0 or missing values in ecosystem C and/or NPP in 3–5 years, we excluded that country. In contrast, if a county had 0 or missing values in ecosystem C and/or NPP in only 1–2 years, we kept that country, but removed all variables for that year(s). The same processing was made for a country with *τ* that was < 0 or > 500 years. Finally, for each country, we reported results from multiple model means. If a country had either “0” or missing values in any of the variables, including ecosystem C storage, NPP, *τ*, *X*_*c*_, and *X*_*p*_ in more than 6 models, we did not include that country. The validations of derived land C sink of top 20 countries were provided in Fig. 1.

The calculations of the yearly average of C pools and NPP of model outputs, regridding of C pools and NPP of model outputs, and sums of C and NPP for countries were performed with the NCAR Command Language (Version 6.6.2, 2019).

## Results

### Global distribution of land carbon sink

**Fig. 2:**
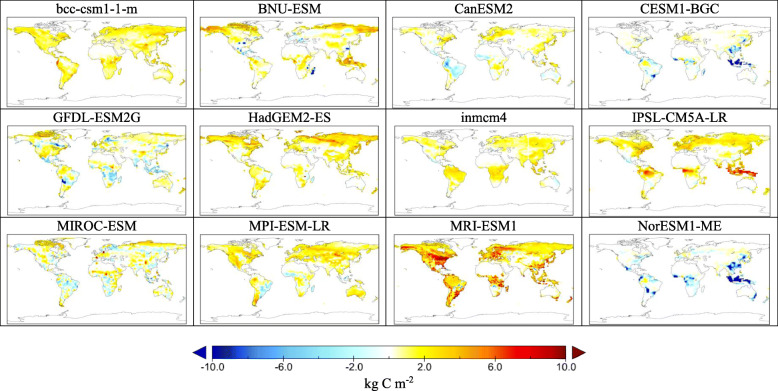
Global maps of land carbon sink by the middle of the 21st century. Shown is cumulative carbon sink by 12 ESMs in CMIP5 over the period from 2005 (average of 2001–2005) to 2050 (average of 2046–2050) under RCP8.5

The global distribution of land C sink shows that most area on Earth will gain C in terrestrial ecosystems under RCP8.5 by the middle of the twenty-first century (Fig. 2). As for the magnitude of C sink, BNU-ESM, HadGEM2-ES, IPSL-CM5A-LR, and MRI-ESM1 predict higher land C sink that other models. Moreover, most ESMs predict that tropical and tundra regions will gain more C than other regions. MRI-ESM1 also predicts greater C sink in northern America and Europe. On the contrary, two ESMs with nitrogen (N) coupled, CESM1-BGC and NorESM1-ME, simulate substantial C loss in tropical region. The C sink in these two models in other regions are also substantially lower than other models.

### Land carbon sink of top 20 countries

**Fig. 3:**
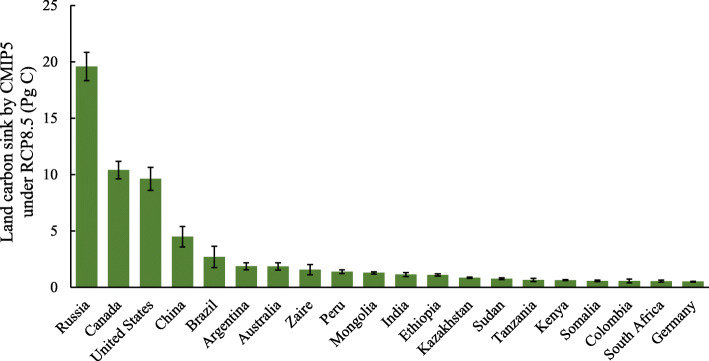
Land carbon sink by the middle of the 21st century of top 20 countries by 12 ESMs in CMIP5 under RCP 8.5 (model means ± SE). Shown is cumulative carbon sink over the period from 2005 (average of 2001–2005) to 2050 (average of 2046–2050)

Unlike the grid level, at the country level, we report results of average land C sink across all CMIP5 models. Land C sink by the middle of the twenty-first century of the top 20 countries is shown in Fig. 3. Total land C sequestered by these 20 countries during 45 years from 2005 to 2050 is 62.1 Pg C. Among them, Russia, Canada, USA, China, and Brazil sequester the most, gaining C of 19.6, 10.4, 9.6, 4.5, and 2.7 Pg C, respectively. These five countries collectively contribute three quarters of the total C sequestrated by the top 20 countries, with Russia sequestering about one third of the total C sink by these 20 countries. However, the rest 15 countries each only gain C less than 2.7 Pg C.

### Attributions of land carbon sink to its causing components

**Fig. 4:**
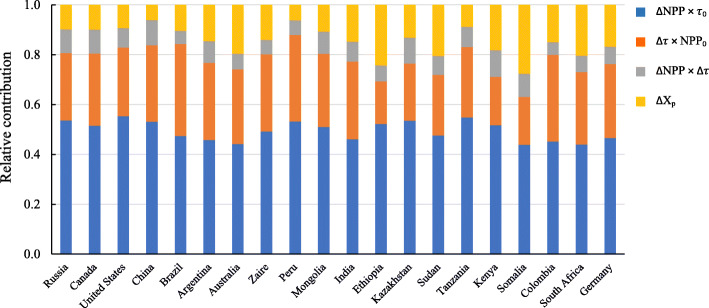
Contributions of each component to cumulative land carbon sink by the middle of the twenty-first century of top 20 countries by 12 ESMs in CMIP5 under RCP 8.5. The four components of land C sink are: production-driven change (∆NPP × *τ*_0_), turnover-driven change (∆*τ* × NPP_0_), interaction between production-driven change and turnover-driven change (∆NPP × ∆*τ*), and change in instantaneous C storage potential (∆*X*_*p*_). NPP_0_ and *τ*_0_ represent NPP and *τ* in year 2005 (average of 2001–2005), respectively. ∆NPP, ∆*τ*, and ∆X_p_ represent changes of NPP, *τ*, and X_p_ between 2050 and 2005 (i.e., average of 2046–2050 minus average of 2001–2005), respectively. NPP: net primary production, *τ*: C residence time, *X*_p_: instantaneous C storage potential

According to the transient traceability framework, land C sink can be decomposed into four components: (1) production-driven change, (2) turnover-driven change, (3) interaction between production-driven change and turnover-driven change, and (4) the change in instantaneous C storage potential. Based on the results of averages across all models, in the four components, production-driven change accounts for the largest proportion, approximately half (49.5%), of the C sink (Fig. 4), followed by turnover-driven change (28.1%), and then the change in instantaneous C storage potential (14.5%). The interaction between production-driven change and turnover-driven change contributes the least to C sink (7.9%).

## Discussion

### Spatial distribution of land carbon sink under the most severe climate change scenario

While there is some uncertainty in simulated land C sink by the CMIP5 models, it is also convergent that terrestrial ecosystems will serve as C sink over the next 30 years in most of the regions on lands as shown in Fig. 2. The two regions that have higher C sink are tropics and tundra, which is also agreed among the models except for the two ESMs with N cycle (CESM1-BGC and NorESM1-ME), in which tropical region will lose C.

Carbon sink of various vegetation types has been investigated at local to regional scales mostly from the perspective of land use change and land use management for mitigating climate change. For example, willow plantation in central Sweden was a sink of ca. 8 ton C ha^−1^ year^−1^, about half of which was attributed to fertilization (Grelle et al. 2007). In Ohio, USA, sequestration rate of soil organic C was estimated to be 62 g C m^−2^ year^−1^ with conversion from conservation tillage to no-till for cultivated Alfisols, and the reforestation of cropland could increase 71 Tg C in 25 years (Tan and Lal 2005). The sequestration potential capacity by afforestation of marginal agricultural land in the Midwestern United State was quantified to be 508–540 Tg C over 20 years and 1018–1080 Tg C over 50 years, which would offset 6–8% of CO_2_ emissions by combustion of fossil fuel in that region (Niu and Duiker 2006). Among the four major plantation crops in tropics, the rubber plantations had much higher C sequestration potential (214 ton C ha^−1^) than Cocoa (65 ton C ha^−1^), orange (76 ton C ha^−1^), and oil palm (45 ton C ha^−1^) plantations (Kongsager et al. 2013). Second-growth forests in the Latin American tropics could potentially accumulate 8.48 Pg C in aboveground biomass over four decades, corresponding to a total sequestration of 31.09 Pg CO_2_ and equivalent to C emissions from fossil fuels and industry by all of Latin America and the Caribbean from 1993 to 2014 (Chazdon et al. 2016).

Globally, most of land C models simulated C sink over the simulation period from 1959 to 2010 (Huntzinger et al. 2017). Cumulative net land C sink was mainly contributed by CO_2_ fertilization and N deposition, whereas climate and land cover change caused C loss from land in most of the models (Huntzinger et al. 2017). For tropic region, climate had a negative effect on land C sequestration, but in northern mid-latitude and arctic-boreal region, it stimulated C sequestration (Huntzinger et al. 2017). Multimodel mean of CMIP5 ESMs showed that land lost 19 Pg C from 1850 to 2005 but with a very wide range across models rooted from the strength of the CO_2_ fertilization effect and differences in model’s implementation of land use change (Jones et al. 2013). However, these models agreed with each other much better on the direction of net land C change in the future; and most of the models predicted a land C sink under four future representative concentration pathways although the magnitude was different among the models (Jones et al. 2013). Similarly, while all eleven coupled climate-carbon cycle models in the C^4^MIP simulated a negative sensitivity for land C cycle to future climate, land will remain as a C sink by 2100 but with a declining magnitude dominated by the reduction of land C uptake in tropics (Friedlingstein et al. 2006).

However, sharing the same land C model (the Community Land Model, CLM4) and having nitrogen cycle incorporated (Thornton et al. 2009), CESM1-BGC and NorESM1-ME simulate a significant C loss in tropical and tundra regions. The C loss is a result of the limited response to increasing atmospheric CO_2_ concentration (Friedlingstein et al. 2014) due to the down regulation by N on photosynthesis (Luo et al. 2004; Friend et al. 2013; Jiang et al. 2015).

### Land carbon sink of different countries under the most severe climate change scenario

Among the top 20 countries with higher land C sink by the middle of the twenty-first century, Russia, Canada, USA, China, and Brazil sequester the most (Fig. 3), which is not surprising in terms of area or location of the countries. These top 20 countries will sequester a substantial amount of C (62.1 Pg C) in their terrestrial ecosystems even under the most severe climate scenario, RCP8.5.

Government policies can have significant effects on anthropogenic GHG emissions. For example, a 17% reduction in daily global CO_2_ emissions during the COVID-19 forced confinement has been reported, with half of reduction resulting from changes in surface transport (Le Quéré et al. 2020). In order to mitigate climate change, countries in the world have long been engaged in efforts to reduce anthropogenic emissions by adopting appropriate policies. In the protocols and agreements envisaged under UNFCCC, many countries have committed to reduce their emissions from fossil fuels and industry with a clear target. However, in these protocols and agreements, C sink by natural ecosystems and its causing components are not well quantified to offset some of the anthropogenic emissions. Globally, anthropogenic CO_2_ emissions from fossil fuels and industry have reached to an average of 9.5 GtC year^−1^ during 2009–2018 (Friedlingstein et al. 2019). China, USA, European Union, and India are the top emitters and collectively account for 59% of global fossil CO_2_ emissions (Friedlingstein et al. 2019).

Land has acted as C sink historically (Huntzinger et al. 2017) and has taken up about one third of fossil CO_2_ emissions (Friedlingstein et al. 2019). Land may keep sequestering C from atmosphere until 2100 as simulated by 8 out of 11 CMIP5 models, but the strength of land C sink becomes weaker and weaker toward the end of the simulation period of 2100 (Friedlingstein et al. 2014). Fully coupled ESMs under the 1pct CO_2_ experiment in CMIP6 also simulate land C sink although the increase of sink declines as terrestrial CO_2_ fertilization effect saturates and the respiratory losses increase as a result of built-up C pools (Arora et al. 2020). For those countries with large areas, the land C sink can be large and offset a substantial proportion of the anthropogenic emissions. For example, in this study, among the four top emitter regions identified in the results by Friedlingstein et al. (2019), USA and China can sequester 9.6 and 4.5 Pg C, respectively by 2050 in their terrestrial ecosystems (Fig. 3), which can contribute to realize their reduction target for mitigating climate change. Federal lands across the conterminous USA will store 19.4% more C in 2050 than in 2005, with forests and grasslands gaining C from 2006 to 2050 at a rate of 620 and 228 kg C ha^−1^ year^−1^, respectively, but shrublands losing C (C sources) at a rate of 13 kg C ha^−1^ year^−1^ (Tan et al. 2015). The C sequestration potential by federal lands in the conterminous USA in the future depends not only on the footprint of individual ecosystems but also on each federal agency’s land use and management (Tan et al. 2015). The estimated ecosystem C sink by evergreen needle-leaved forests in China increased rapidly with age, reached peak value of 0.45 kg C m^−2^ year^−1^ at age of 22 years, and decreased gradually after that (Zhou et al. 2015). The highest C sink efficiency (i.e., C sink per unit NPP) of these evergreen needle-leaved forests occurred when forest age was between 11 and 43 years.

### Components that control land carbon sink

The transient traceability framework allows us to quantify not only the land C sink of different countries but also the relative contributions of the involving processes, including production-driven change, turnover-driven change, interaction between production-driven change and turnover-driven change, and the change in instantaneous C storage potential. Among these four components, production-driven change accounts for the largest proportion of the C sink (Fig. 4). The second important component is turnover-driven change; and then the third one is change in instantaneous C storage potential. The interaction between production-driven change and turnover-driven change accounts for the least to C sink.

Carbon stored in terrestrial ecosystems is not only determined by how much C enters the ecosystem but also determined by how long C will stay in that system, with the former being known as NPP and the latter as C residence time. NPP is usually high in tropical regions and low in high latitude (Todd-Brown et al. 2013). Rising CO_2_ in atmosphere favorites NPP, which is well known as CO_2_ fertilization. The fertilization of CO_2_ on NPP, including both aboveground NPP (ANPP) and belowground NPP (BNPP), has been extensively demonstrated in manipulative experiments with elevated CO_2_ conducted in dozens of ecosystems (Song et al. 2019). Models also consistently simulate the fertilization of CO_2_ with a multiple model mean for carbon-concentration feedback parameter *β* being 0.92 Pg C ppm^−1^ (Arora et al. 2013). As a result of CO_2_ fertilization, cumulative net land C sink over the period 1959 to 2010 is overwhelmingly contributed by atmospheric CO_2_, especially for tropics and extratropics (Huntzinger et al. 2017). Global NPP will keep increasing with time till 2100 because of rising CO_2_ (Friend et al. 2013)

Changes in precipitation have profound impacts on NPP. Increased precipitation usually enhances NPP and decreased precipitation reduces NPP, with ANPP being more sensitive than BNPP (Wilcox et al. 2017; Song et al. 2019). However, the sensitivity of ANPP to precipitation change would be saturating, likely driven by ANPP responses to extreme precipitation (Knapp et al. 2017; Wilcox et al. 2017). Primary production has been found to be most sensitive to precipitation in dryland and grassland ecosystems (Maurer et al. 2020). The trend and interannual variability of the global land C sink are dominated by semi-arid ecosystems where variations in precipitation and temperature strongly regulate ecosystem C balance (Ahlström et al. 2015).

Climate warming, however, has varied effects on NPP. Earth system models involved in CMIP5 simulate negative effects of warming on land C fluxes, with the multiple model mean for carbon-climate feedback parameter *γ* of − 58.4 Pg C °C^−1^ (Arora et al. 2013). In a recent synthesis of the manipulative experiments of warming in the world, NPP and ANPP both remain unaltered but BNPP is slightly increased by warming (Song et al. 2019). In contrast, Wu et al. (2011) concluded that experimental warming stimulates NPP in another global synthesis with 85 studies. In a 13-year field warming experiment in a tallgrass prairie in Oklahoma, USA, warming consistently increases ANPP and BNPP, and the increased ANPP and BNPP are positively correlated with the proportion of ANPP contributed by C_3_ forbs (Xu et al. 2015).

Observational data and model simulations both show that C residence time is strongly dependent on temperature, that is, longer in colder regions than in warmer regions, with tundra and boreal forests having longest C residence time than other terrestrial ecosystems (Carvalhais et al. 2014). Consistent with the relationship between C residence time and temperature, climate warming has been demonstrated to stimulate decomposition and therefore shorten C residence time, especially in cold regions (Friend et al. 2013; Tian et al. 2015). However, the widely accepted relationship between C residence time and warming may not hold true when accounting for changes in C age structure and composition of respired C as found by Lu et al. (2018). In their study, warming can cause an increase in global C residence time due to the depletion of fast-turnover C pool and accompanied changes in compartment C age structures.

Carbon residence time has a strong association with precipitation in the observation-based data set, which is surprising, but the pattern is not well reproduced by those CMIP5 models, highlighting that the hydrological cycle could be more important in affecting C cycle than it is represented in model simulations (Carvalhais et al. 2014). Rising atmospheric CO_2_ and N deposition help shorten residence time of soil C (Tian et al. 2015). The decreased ecosystem C residence time under elevated CO_2_ might be a result of replenishment of C into fast turnover C pool and subsequent decrease in compartment C age structure (Lu et al. 2018). A recent study with a global land surface model indicates that ecosystem C residence time has been reduced from 74 years in the 1860s to 64 years in the 2000s, due mainly to land use change and climate change (Wu et al. 2020). For soil C, however, land use change can bring either increased or decreased residence time of soil C (Tian et al. 2015). Predicted vegetation C residence times under future CO_2_ and climate are increased, decreased, or stable, depending on different regions and due to changes in tree mortality and composition of vegetation types (Friend et al. 2013).

Conclusions on the relative importance of NPP and C residence time in determining land C storage vary with different C pools. Vegetation C storage is determined more by C residence time than NPP (Friend et al. 2013; Jiang et al. 2015). For soil C storage simulated by 11 ESMs in CMIP5, NPP and soil temperature explain much of the spatial variations in soil C (Todd-Brown et al. 2013). As for ecosystem C storage, more than half of land C storage (~ 60%) is determined by ecosystem baseline residence time (Zhou et al. 2021).

Factors controlling changes in C storage, that is, C sink or source, might be different from those controlling transient C storage. The domination of productivity-driven changes (i.e., ∆NPP × *τ*_0_ in this study) over turnover-driven changes (i.e., ∆*τ* × NPP_0_ in this study) in controlling land C pool changes has been detected by Koven et al. (2015) for both live C pools and dead C pools. This is understandable from mathematical perspective because turnover time is usually 0–48 years and NPP is 0–2 kg C m^−2^ year^−1^ (Koven et al. 2015). However, less control of turnover-driven change compared to productivity-driven change on C pool changes in response to the imposed forcings may result from the lack of process representation behind the changing turnover times, such as allocation and mortality for live C pool; and permafrost, microbial dynamics, and mineral stabilization for dead C pool (Koven et al. 2015).

While the contributions by the changes in instantaneous C storage potential, *X*_*p*_, is not much, it can help bring 14.5% of C sink. The instantaneous C storage potential, *X*_*p*_, is potential C sequestration restricted otherwise by environmental factors and other factors such as disturbances (Luo et al. 2017). Our results imply that if we manage our terrestrial ecosystems to the best conditions for the ecosystems, the ecosystems can store 14.5% more C. The proportion of instantaneous C storage potential in simulated global transient land C storage by 7 CMIP6 ESMs is 4.5% (Zhou et al. 2021), but its contributions in C sink has been amplified by 3 times. Accounting for only 7.9% of land C sink, the interaction between production-driven change and turnover-driven change (i.e., ∆NPP × ∆*τ*) represents the interaction between change in NPP and change in C residence time. Our results demonstrate that change in NPP and change in C residence time are interactive in determining C sink.

In most previous studies that investigated the role of C residence time, a steady-state assumption has been applied, in which C residence time is derived by C storage divided by C inputs, GPP or NPP. This can bring significant bias in estimating ecosystem residence time (Lu et al. 2018). The transient traceability framework allows us to disaggregate the individual components of land C sink of different countries, which eradicates the bias rooted in the steady-state assumption in C residence time. Due to the challenge to apply the full transient traceability framework to CMIP5 model outputs (Jiang et al. 2017), we are not able to further decompose C sink into more specific C processes in this analysis. In the future, with more detailed information of those models, we may decompose C sink of different countries further and can thus know how individual C processes regulate land C sink better. Finally, as acknowledged by Koven et al. (2015), CMIP5 models may have some fundamental bias as reflected by the large uncertainty in the simulations across CMIP5 models shown both in this analysis and in many previous studies, which is a major challenge in predicting land carbon dynamics. To eliminate any bias related to selections of individual models, as a common practice in analyzing model results, we used the means of multiple models instead of results of any single model to represent the best model simulation results. Even though the use of the multiple model means to best represent the CMIP5 model simulation results in this analysis, the uncertainty across these CMIP5 models should be taken into account and needs to be carefully evaluated when making policies.

## Conclusions

Our analysis of CMIP5 results suggests that most areas in the world will act as land C sink by the middle of the twenty-first century under RCP8.5, especially in tropical and tundra regions. The top 20 countries with the highest C sink can sequester 62.1 Pg C in total, with Russia, Canada, USA, China, and Brazil sequestering the most and collectively accounting for three quarters of the total C sequestrated by the top 20 countries. Among the four traceable components of land C sink, production-driven change contributes the most, approximately half, highlighting the joint determinations of land C sink by change in NPP and inherent C residence time of the countries. Turnover-driven change is the second largest component of land C sink, which indicates that original ecosystem NPP and change in C residence time also play a relative important role for terrestrial ecosystems to gain C. Better management of the terrestrial ecosystems can also help realize the maximal land C sink while the change in instantaneous C storage potential, *X*_*p*_, contributes a small proportion of C sink. Overall, land C sink from the terrestrial ecosystems can offset a substantial proportion of greenhouse-gas emissions, which should be better accounted in the future agreements by the United Nations Framework Convention on Climate Change.

## Data Availability

The CMIP5 data used in this study is available through the Earth System Grid Federation (ESGF): http://esgf-node.llnl.gov/. The data for the figures in this study is available at the authors’ website: http://www2.nau.edu/luo-lab/download.

## Abbreviations

C: Carbon
CMIP5: Coupled Model Intercomparison Project Phase 5
СОР: Conference of the Parties
ESM: Earth system models
N: Nitrogen
NEE: Net ecosystem exchange
NEP: Net ecosystem production
NPP: Net primary production
RCP: Representative concentration pathway
UNFCCC: United Nations Framework Convention on Climate Change
